# The loose evolutionary relationships between transcription factors and other gene products across prokaryotes

**DOI:** 10.1186/1756-0500-7-928

**Published:** 2014-12-17

**Authors:** Marc del Grande, Gabriel Moreno-Hagelsieb

**Affiliations:** Department of Biology, Wilfrid Laurier University, 75 University Ave. W, N2L 3C5 Waterloo, Ontario Canada

**Keywords:** Transcription factors, Interactome, Evolvability, Comparative genomics, Phylogenetic profiles, Regulatory interactions

## Abstract

**Background:**

Tests for the evolutionary conservation of associations between genes coding for transcription factors (TFs) and other genes have been limited to a few model organisms due to the lack of experimental information of functional associations in other organisms. We aimed at surmounting this limitation by using the most co-occurring gene pairs as proxies for the most conserved functional interactions available for each gene in a genome. We then used genes predicted to code for TFs to compare their most conserved interactions against the most conserved interactions for the rest of the genes within each prokaryotic genome available.

**Results:**

We plotted profiles of phylogenetic profiles, p-cubic, to compare the maximally scoring interactions of TFs against those of other genes. In most prokaryotes, genes coding for TFs showed lower co-occurrences when compared to other genes. We also show that genes coding for TFs tend to have lower Codon Adaptation Indexes compared to other genes.

**Conclusions:**

The co-occurrence tests suggest that transcriptional regulation evolves quickly in most, if not all, prokaryotes. The Codon Adaptation Index analyses suggest quick gene exchange and rewiring of transcriptional regulation across prokaryotes.

**Electronic supplementary material:**

The online version of this article (doi:10.1186/1756-0500-7-928) contains supplementary material, which is available to authorized users.

## Background

Using data derived from literature on experimentally determined molecular interactions, previous work suggested that gene and gene product relationships brought about through transcriptional co-regulation in *Escherichia coli* K12 MG1655, have loose evolutionary conservation [1–3]. Such results suggest that transcriptional regulation might evolve quickly, an idea that gains support from other results, such as those suggesting that at least half of the transcription factors (TFs) present in *E. coli* might come from horizontal gene transfer [4].

Profiles of phylogenetic profiles, p-cubic, can provide information about the quality of functional interaction datasets [5], and about the evolutionary conservation of known functional interactions [3]. As mentioned in the paragraph above, the work on evolutionary conservation has relied on experimentally determined interactions, such as those gathered in knowledge databases like RegulonDB [6] and EcoCyc [7]. Knowledge databases are not readily available for most other genomes. It is therefore not possible to further test previous results in other genomes in the same way. While TFs might be determined by the presence of DNA-binding domains in encoded proteins, their target genes, for example, would not be known. However, the co-occurrence across genomes of genes coding for TFs with other genes can be measured, and we reasoned that maximally co-occurring genes might still reflect the evolutionary stability of interactions between TFs and other genes in any given genome (see further explanations under Results and discussion).

In this work we show evidence suggesting that the most evolutionarily conserved interactions for the sets of predicted TFs across a wide sample of publicly available prokaryotic genomes are less conserved than the most conserved interactions among all other gene products.

## Methods

### Genomes and phylogenetic profiles

Using a web-based tool [8], we selected a non-redundant genome dataset filtered using a genomic similarity score [3, 8, 9] chosen to keep the equivalent of one genome per represented species (*GSSa*=0.90) out of the 2733 prokaryotic genomes available at the RefSeq database [10] (ftp://ftp.ncbi.nih.gov/genomes/Bacteria/) by the end of December 2013. We further filtered this non-redundant genome dataset to keep genomes longer than 2.5 Mbp, with at least 80 genes coding for transcription factors other than sigma factors.

To build phylogenetic profiles, we used NCBI’s *blastp* [11] to determine orthologs as reciprocal best hits (RBHs) as described previously [12, 13]. Each of the non-redundant genome datasets above was compared against a non-redundant genome dataset filtered at a *GSSa* threshold of 0.75 to build phylogenetic profiles, a threshold previously shown to produce phylogenetic profiles with good discrimination between genes coding for functionally interacting proteins and genes coding for non-interacting proteins [9]. Presence of a RBH was represented with 1, absence with 0. We used mutual information (MI), measured in bits, to compare the similarity of phylogenetic profiles [9, 14]. The formula for MI is:
1

Profiles of phylogenetic profiles (p-cubic) [3, 5], are graphs representing the proportion of genes left at different thresholds of MI. Briefly, these graphs are anti-cumulative plots showing the decline in the proportion of pairs of genes left at increasing MI thresholds. Pairs of genes whose interactions are highly conserved should have higher MI than those with poorly conserved ones. Therefore, if a group contains a higher proportion of highly conserved interactions, their p-cubic line should tend to drop at a slower rate than the p-cubic of a group with less conserved interactions [see Figure one in [3]]. These graphs are very similar in concept to those presented previously by Date and Marcotte [15].

### Transcription factors

Experimentally determined TF datasets were obtained as follows: for *Escherichia coli* strain K12-MG1655 we downloaded the lists of predicted and manually curated TFs from RegulonDB [6], for *Bacillus subtilis* strain 168 we downloaded the lists of TFs from the DBTBS [16].

To identify TFs in the rest of the genomes in our study, we downloaded the lists of TF-related Pfam and Superfamily identifiers from the DNA-Binding Domains database (DBD) [17]. We then ran *hmmer* (version 3.1b1) [18] comparisons of all the annotated proteins for each genome against the Hidden Markov Models of these domain families. The domain families were extracted with the *hmmfetch* program from the *Pfam-A.hmm* file from the Pfam database (version 27, http://pfam.janelia.org/) [19], and from the Superfamily *hmmlib* file (version 1.75; http://supfam.cs.bris.ac.uk/SUPERFAMILY/downloads.html) [20]. For Pfam families we used the *hmmscan--cut_ga* option which uses the “gathering threshold” set for each family in Pfam. For Superfamily we downloaded as many pre-annotated genomes as available at the database. For other genomes we set an *hmmscan* maximum domain e-value of 0.0001 (*--domE 1e-4*), then filtered out the *hmmer* results using the *ass3.pl* script available from the Superfamily download site. We ensured that this procedure was adequate by running a couple of the pre-annotated genomes and verifying that we had the very same results.

### Evaluation of p-cubic differences

To evaluate whether the p-cubic curves for non TF-coding genes were above or below the p-cubic curves for TF-coding genes within each organism, we divided the curves into bins and calculated the difference between the non-TF bin and its corresponding TF bin. The number of bins (*n* in the equation below) was set to 20, because we found that, in most of the genomes analyzed, bins thus produced had enough data for the operations. We normalized this value by the total number of bins. This operation yielded what we call the delta p-cubic:
2

Values of *ΔP*3 > 0 indicate that the p-cubic curve for TF-coding genes fell below the curve for non TF-coding genes, indicative of TFs forming looser associations than other gene products. Values of *ΔP*3 ≤ 0 indicate either equal association strength (*ΔP*3 = 0), or TF-coding genes forming stronger associations (*ΔP*3 < 0). After filtering, this analysis was performed on 790 prokaryotes, including some Archaea.

### Codon Adaptation Index

We determined the Codon Adaptation Index (CAI) [21] for each gene within each of the non-redundant genomes chosen above (0.90 *GSSa*). The CAI compares the codon usage of a protein-coding gene against the codon usage of highly expressed genes (HXGs). The best examples of HXGs are those coding for ribosomal proteins. To find ribosomal proteins we used the COG and arCOG ribosomal protein families described by Yutin *et al.* [22]. These COGs and arCOGs were matched to their corresponding bacterial and archaeal genomes and gene identifiers using the files provided by the authors (ftp://ftp.ncbi.nih.gov/pub/wolf/COGs/). If a genome in our database was not in those files, we checked the COG annotations provided with the genomes as downloaded from NCBI and/or compared, using the *rpsblast* program (part of NCBI’s BLAST+ suite) [11], the encoded proteins of each genome to the profiles for the appropriate COGs found at the Conserved Domains Database database [23]. The *rpsblast* program was run with soft-masking (*-seg yes -soft_masking true*), a Smith-Waterman final alignment (*-use_sw_tback*), and a maximum e-value threshold of 1×10^-6^ (*-evalue 1e-6*).

To calculate the codon usage tables of the HXGs (the ribosomal protein-coding genes chosen above) of each genome, we used the program *cusp* from the EMBOSS software suite [24]. We then used these HXGs codon usage tables to calculate the CAI for each protein-coding gene within the appropriate genome using the *cai* program also from the EMBOSS software suite.

## Results and discussion

### Top-scoring interactions in model organisms suggest that both experimentally-known and predicted TFs have less conserved interactions than other genes

Ideally, we would analyze the p-cubic of TFs and the genes in their target transcription units. However, databases containing enough literature-based data exist only for a few model organisms. Therefore, we developed computational strategies for gathering data of sufficient quality to perform these analyses across available genomes. We needed two kinds of data: (a) TFs and (b) their target genes.

Our strategy towards finding TFs consisted of downloading manually curated datasets [6, 16], predictions produced by other authors [6, 16, 20], as well as comparing the annotated proteins of each genome against previously described DNA-binding Pfam and Superfamily domains as described in the DBD database [17]. We kept only the genomes containing at least 80 predicted TFs, where predicted TFs were genes coding for proteins matching the Pfam and Superfamily domains listed at the DBD database. This procedure reduced our prokaryotic non-redundant set from 950 to 857 (we provide tables of predicted TFs across the full set of 950 genomes used in this study as Additional files 1 and 2).

Once we found putative TFs in the genomes of interest we still needed target genes (TGs). Properly finding TGs can be quite a demanding task. However, we thought that we could still compare the conservation of more generic TF associations, not necessarily a TF gene to TG association, against the conservation of other gene associations. To this end we used mutual information (MI) as a measure of co-occurrence. We calculated the MI for every pair of genes in each of the 750 genomes selected above. For each gene, we selected its five top-scoring pairs as representatives of its most conserved interactions. For example, in *E. coli* K12, the gene *lptD* (gi|16128048) shows MI values against the other 4138 protein-coding genes in this genome ranging from 0 to 0.51, with the five top-scoring ones being 0.43, 0.44, 0.46, 0.48, and 0.51. We used these top-scoring values as representatives for the most evolutionarily conserved interactions of this gene.

**Figure 1:**
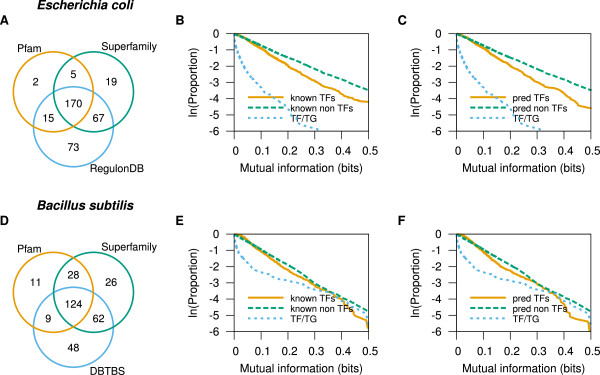
**Comparing manually curated, and predicted transcription factors (TFs).** Manually-curated TFs were obtained from RegulonDB [6] for *E. coli* K12, and from DBTBS [16] for *B. subtilis* 168. Predictions were based on matches to Pfam and Superfamily DNA-binding domains reported at the DBD [17]. **(A, D)** Venn diagrams comparing curated and predicted TFs. **(B, E)** P-cubic of curated TFs. **(C, F)** P-cubic of predicted TF-coding genes. The p-cubic of any set of TF-coding genes is below the p-cubic of the corresponding set of other genes, showing that TF-coding genes have lower co-occurrences than other genes. Since predicted TF-coding genes behave similarly to curated TF-coding genes, predicted TF-coding genes might be enough to test if TF-coding genes in other prokaryotes have lower co-occurrences than genes coding for proteins other than TFs.

In our two model organisms, *E. coli* K12 MG1655 and *B. subtilis* 168, the highest MI of genes coding for manually-curated TFs shows a lower p-cubic than that for other genes (Figure 1B and 1E). The same was true when we used the genes coding for predicted TFs in the same genomes (Figure 1C and 1F). This suggests that, even though our predicted TFs do not completely agree with the curated datasets (Figure 1A and 1D), they still provide enough information to test the conservation of TF interactions against the interactions of other genes. Our previous study on the evolutionary conservation of functional interactions of *E. coli* K12 had found that the most conserved regulon-related interaction was between TFs and their TGs [3]. Here we found that the top-scoring interactions for TFs have better conservation than the TF to TG interactions (Figure 1). Being a rather small set, the p-cubic curves for known TF/TG pairs is too noisy to allow confident conclusions. Still, it is possible that top-scoring interactions represent interactions beyond those mediated by transcriptional regulation. However, top-scoring interactions for TFs were still lower than those for other genes, suggesting that TFs have more generic evolutionary plasticity than other genes in these model organisms.

The MI for both predicted and experimentally validated TFs from *B. subtilis* does not show as strong a difference to other genes as they do in *E. coli*. We do not have a full explanation for this difference in results. It could be that the interactions between TFs and target genes in *B. subtilis* is closer in evolutionary conservation to those of other genes. It could also be that the genomes in the database do not represent enough information to show the difference with enough emphasis. Finally, gene over-annotations in *B. subtilis*, as estimated by the SwissProt method proposed by Skovgaard *et al.* [25, 26], is higher for *B. subtilis* (16.6%) than for *E. coli* (5.32%). False genes would necessarily have no orthologs in other genomes, and their MI with other genes would necessarily be zero. Thus, false genes might lower the p-cubic curve of genes other than those coding for TFs (genes coding for TFs would most probably be true genes because their products match true protein domains).

### Top-scoring interactions suggest that TFs have less conserved interactions than other genes among prokaryotes

The results above show p-cubic comparisons suggesting that TFs have less co-occurring, and therefore less conserved, interactions than other genes in model organisms (Figure 1). Since predicted TFs produced similar results to those obtained with manually-annotated TFs, we concluded that genes coding for predicted TFs in other prokaryotes would yield appropriate results to evaluate if TF-coding genes also show a tendency towards less evolutionarily conserved interactions than other genes in other prokaryotes.

To test for the conservation of functional associations between TF-coding genes and other genes in prokaryotes other than model organisms, we selected genomes at least 2.5 Mbp in length from NCBI’s RefSeq database (see Methods). We filtered small genomes because it is well known that prokaryotes with reduced genomes tend to lack TF-coding genes [27–29]. We also filtered out redundant genomes using a previously published method to cluster similar genomes and keeping only one as a representative [8], and rejected genomes with less than 100 genes coding for predicted TFs (see Methods).

**Figure 2:**
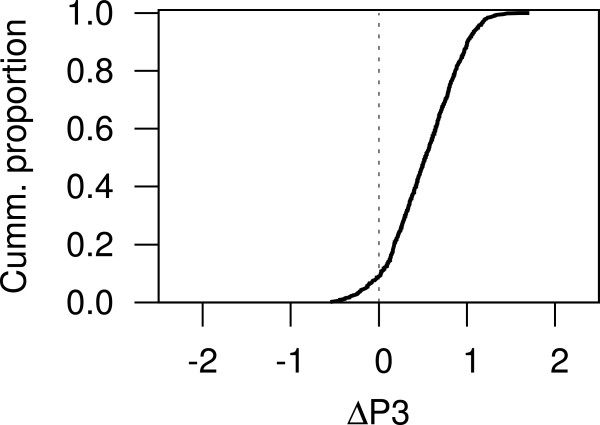
***Δ*** **P3 between predicted TF-coding genes and other genes across the prokaryotic genomes analyzed in this study.** If the p-cubic curve for genes coding for proteins other than TFs runs above the p-cubic curve for TF-coding genes the *Δ*P3 will be positive. Thus, a positive *Δ*P3 indicates less evolutionarily conserved interactions for TF-coding genes. A negative *Δ*P3 would indicate the opposite. The cumulative proportion shown here indicates that TF-coding genes in approx. 91% of the genomes tested have less conserved interactions than other genes.

To summarize the results for each of the genomes chosen above, we calculated a difference, *Δ*P3, between the p-cubic curve for genes other than predicted TFs and the p-cubic for predicted TFs (see Methods). A *Δ*P3 above zero would indicate that the p-cubic for TF-coding genes shows lower co-occurrence than the p-cubic of other genes, while a *Δ*P3 below zero would indicate that TF-coding genes have a higher tendency to co-occur, and therefore contain more evolutionarily conserved interactions than other genes. The cumulative curve of *Δ*P3s shows that genes coding for predicted TFs have less co-occurrence, and therefore proportionally fewer conserved interactions than other genes in 780 of the 857 genomes tested (91%; Figure 2), thus confirming that TF interactions might evolve quickly in most, if not all prokaryotes.

### Low CAIs suggest that genes coding for TFs tend to be horizontally transferred

**Figure 3:**
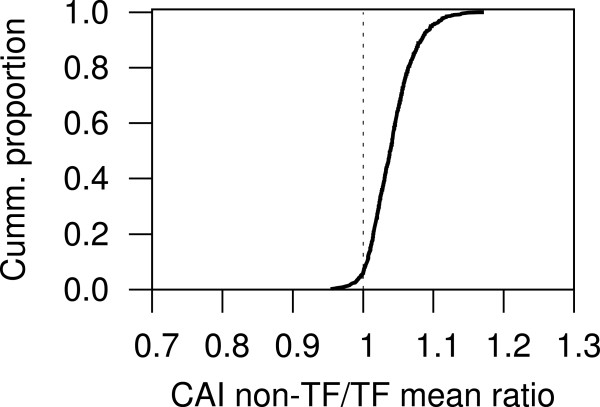
**Comparing the Codon Adaptation Index of predicted transcription factor-coding genes and other genes across prokaryotic genomes.** This curve shows that TF-coding genes in approx. 94% of the genomes tested had lower average CAI than other genes, thus suggesting that TF-coding genes tend to be more horizontally transferred than other genes.

Previous work has suggested that at least half of the TF-coding genes of *E. coli* come from horizontal gene transfer (HGT) events [4]. This might be one of the reasons why associations brought about via TFs evolve quickly (another reason might be that operators, the sites in DNA where TFs bind, have low information contents, meaning that they can easily evolve [30]). To further test for the possibility of TF-coding genes coming from HGT across prokaryotes we calculated the Codon Adaptation Index (CAI) for all the genes of the genomes under analysis. We found that the CAI of TF-coding genes tends to be lower than that of non-TF-coding genes in 809 of the 857 (94%) of the genomes containing at least 80 predicted TFs (Figure 3). Furthermore, t-tests showed significant differences between the CAIs of non-TFs and TFs in 691 of the 857 genomes (80%), out of which 676 (98%) had a positive statistical difference (p ≤ 0.05; see Additional files 1 and 2). Our results are also in agreement with previous work showing that genes predicted to have been horizontally transferred are enriched in genes encoding for proteins with DNA-binding functions [31].

While CAI alone might be insufficient for determining HGT [32–34], our results still suggest that TF-coding genes might be frequently transferred among prokaryotes.

## Conclusion

In this work we presented data across several prokaryotic genomes suggesting that genes coding for TFs have evolutionarily loose relationships with other genes, and that genes coding for TFs have a tendency towards having low Codon Adaptation Indexes compared to other gene sets, suggesting that TF-coding genes are frequently horizontally transferred. Overall, these results suggest that transcriptional regulation evolves quickly among prokaryotes, and that the evolution of transcriptional regulation might be strongly tied to elements specializing in horizontal gene transfer, like pathogenicity and other genomic islands. It is therefore tempting to hypothesize that genomic islands might be of main importance in the evolution of transcriptional regulation.

## Availability of supporting data

We provide predicted transcription factors across prokaryotic genomes used in this study at: http://microbiome.wlu.ca/TFs/

## Electronic supplementary material

Additional file 1: ***Δ*** **P3 stats table.** Table with *Δ*P3 calculations for all genomes available in the study. (ZIP 29 KB)

Additional file 2: **CAI stats table.** Table with codon adaptation index calculations for all genomes available in the study. (ZIP 20 KB)
